# Real-World Experience with Non-Metastatic Male Breast Cancer: A 222-Patient Multicenter Study from the Turkish Oncology Group (TOG)

**DOI:** 10.3390/cancers17243895

**Published:** 2025-12-05

**Authors:** Ülkü Yalçıntaş Arslan, Ferit Aslan, Murat Ayhan, Nadiye Akdeniz, Gözde Tahtacı, Havva Yeşil Çınkır, Mevlude İnanç, Gökşen İnanç İmamoğlu, Necati Alkış, Mustafa Başak, Nuriye Özdemir, Muhammet Ali Kaplan, Ömür Berna Öksüzoğlu

**Affiliations:** 1 Medical Oncology, Dr. Abdurrahman Yurtaslan Ankara Oncology Research and Training Hospital, 06200 Ankara, Türkiye; ulkuarslan63@gmail.com (Ü.Y.A.);; 2 Medical Oncology, Yüksek İhtisas University Affiliated Medicalpark Ankara Batıkent Hospital, 06370 Ankara, Türkiye; 3 Medical Oncology, Kartal Dr. Lütfi Kırdar Research and Training Hospital, Liv Vadi Hospital, 34475 İstanbul, Türkiye; muratayfaruk@gmail.com; 4 Medical Oncology, Dicle University Faculty of Medicine, 21280 Diyarbakır, Türkiye; nadiyeakdeniz21@gmail.com (N.A.);; 5 Medical Oncology, Gazi University Faculty of Medicine, 06170 Ankara, Türkiye; drgozdetahtaci@gmail.com (G.T.);; 6 Medical Oncology, Gaziantep University Faculty of Medicine, 27310 Gaziantep, Türkiye; drhavva1982@gmail.com; 7 Medical Oncology, Erciyes University Faculty of Medicine, 38039 Kayseri, Türkiye; 8 Medical Oncology, Etlik City Hospital, 06200 Ankara, Türkiye; 9 Medical Oncology, Kartal Dr. Lütfi Kırdar Research and Training Hospital, Medicalpark Tokat Hospital, 60230 Istanbul, Türkiye

**Keywords:** male breast cancer, non-metastatic breast cancer, prognostic factors, disease-free survival, overall survival, hormone receptor–positive disease, HER2-positive breast cancer

## Abstract

**Background/Objectives:** Male breast cancer (MBC) is rare, and evidence guiding adjuvant treatment remains limited due to the absence of randomized trials. Most therapeutic strategies are extrapolated from female breast cancer data. This multicenter study evaluated real-world treatment patterns, prognostic factors, and long-term outcomes in men with non-metastatic MBC in the modern systemic therapy era. **Methods:** Clinical data from 222 men with stage I–III breast cancer diagnosed between 1986 and 2018 across eight oncology centers were retrospectively analyzed. Survival outcomes were estimated using Kaplan–Meier methods, and prognostic factors were evaluated using Cox proportional hazards models. **Results:** The median age was 61 years; most patients had invasive ductal carcinoma (91.9%) and hormone receptor-positive disease (91.8%). Axillary lymph node involvement was present in 57.7%, and 30.6% had T3–T4 tumors. The median follow-up was 59.5 months. Recurrence occurred in 36.9% of patients and 35.6% died. Median disease-free survival (DFS) and overall survival (OS) were 77 and 119 months, respectively. Advanced tumor stage significantly worsened DFS (HR: 1.82; 95% CI: 1.23–2.69) and OS (HR: 1.74; 95% CI: 1.15–2.64). Lymph node positivity was a strong predictor of inferior DFS (HR: 2.41; 95% CI: 1.62–3.59) and OS (HR: 1.89; 95% CI: 1.20–2.98). High-grade tumors further increased the risk of recurrence (HR: 2.27; 95% CI: 1.44–3.58) and mortality (HR: 1.96; 95% CI: 1.19–3.21). In HER2-positive patients, recurrence was lower among those receiving adjuvant trastuzumab (25.0% vs. 52.4%). Although adjuvant chemotherapy did not improve survival in the overall cohort, it significantly benefited node-positive patients (DFS HR: 0.58; 95% CI: 0.37–0.90; OS HR: 0.53; 95% CI: 0.32–0.88). **Conclusions:** Despite the widespread use of modern systemic therapies, classical prognostic markers—including tumor size, nodal status, and histologic grade—remain the strongest determinants of survival in men with breast cancer. These findings underscore the importance of early diagnosis and highlight the need for male-specific prospective studies.

## Simple Summary

Male breast cancer is a rare disease, and most treatment decisions are based on data from female patients. In this large multicenter study from Turkey, we evaluated 222 men with non-metastatic breast cancer treated over more than three decades. We found that tumor size, lymph node involvement, and tumor grade were the strongest predictors of survival. Adjuvant chemotherapy significantly improved outcomes in high-risk patients, especially those with lymph node-positive or high-grade tumors, while trastuzumab was beneficial for HER2-positive disease. Despite advances in therapy, many men were still diagnosed at an advanced stage, highlighting the need for greater awareness and earlier detection. Our findings provide real-world evidence that can help guide treatment decisions in this rare patient population.

## 1. Introduction

Male breast cancer (MBC) is a rare disease, representing approximately 1% of all breast cancers worldwide [1,2]. A large Turkish dataset including 13,079 patients diagnosed between 1992 and 2017 reported an incidence of 1.3%, slightly higher than global estimates [3]. While MBC remains uncommon, its incidence has shown a gradual increase, whereas mortality has steadily declined in recent years in the United States [4]. Nevertheless, a SEER-based analysis demonstrated that although survival improved significantly for both sexes between 1976 and 2005, the improvement was more pronounced in women, indicating persistent sex-related disparities [5].

MBC is predominantly characterized by hormone receptor positivity, with estrogen receptor (ER) expression detected in the majority of cases [6]. Historically, tamoxifen monotherapy constituted the standard adjuvant treatment in MBC [7]. Early analyses by Cutuli reported that adjuvant chemotherapy (CT) was used in only 7.5–29% of male patients [8], increasing to 37% by 2010 in updated cohorts from the same group [9]. More contemporary U.S. data show that the adjuvant CT rate has risen to 45.5% among stage I–III MBC cases diagnosed between 2004 and 2016 [10], reflecting a shift toward adopting female breast cancer treatment standards.

In a large cohort with centralized pathological review, ER positivity reached 99.3%, whereas HER2 positivity was identified in only 8.7% of patients diagnosed between 1990 and 2010 [11]. In this study, 76.8% of non-metastatic patients received adjuvant endocrine therapy primarily tamoxifen while nearly one third received neoadjuvant or adjuvant CT [12]. Evidence regarding older regimens such as CMF remains limited and is based on a small prospective trial with long-term results accessible since 2006 [12,13]. Despite increasing application of modern systemic therapies, treatment approaches for MBC continue to rely largely on extrapolation from female breast cancer studies due to the absence of male-specific randomized clinical trials.

We previously reported outcomes from 113 men diagnosed with non-metastatic MBC, demonstrating that tumor size and lymph node involvement were the most important prognostic factors, despite 73.7% of the cohort receiving adjuvant CT [14]. Given the substantial evolution in systemic therapy over the past three decades, updated real-world data are essential. The objective of the present study was to reassess survival outcomes, treatment patterns, and prognostic determinants in men with non-metastatic MBC diagnosed between 1986 and 2018 across multiple centers.

## 2. Materials and Methods

### 2.1. Study Design and Patient Selection

This multicenter retrospective cohort study included male patients diagnosed with non-metastatic (stage I–III) breast cancer between January 1986 and December 2018 across eight oncology centers in Turkey. Eligible patients were identified through institutional databases, pathology archives, and electronic medical records. Inclusion criteria were: histologically confirmed invasive breast carcinoma, male sex at birth, absence of distant metastasis at diagnosis, availability of detailed clinicopathological and treatment data, and at least 12 months of follow-up unless relapse or death occurred earlier. Patients with de novo metastatic disease, carcinoma in situ without invasion, missing essential information, or inadequate follow-up were excluded. Because complete archival case lists were unavailable for certain early years of the study period, the exact number of initially screened patients could not be determined. Therefore, only the final eligible cohort (*n* = 222) is reported, and all exclusion criteria are specified without numerical breakdown. A total of 222 patients met these criteria and were included in the final analysis.

### 2.2. Data Collection and Variables

Clinical, pathological, and treatment-related data were extracted retrospectively from institutional archives using standardized data collection forms. Variables included demographic characteristics (age at diagnosis, comorbidities), tumor features (histologic subtype, grade, laterality, TNM stage, ER/PR/HER2 status), surgical procedures, use and type of adjuvant or neoadjuvant chemotherapy, endocrine therapy, radiotherapy, and HER2-directed treatments, as well as follow-up information including dates and sites of recurrence, survival status, and cause of death where available. Pathology assessments were performed according to institutional standards valid at the time of diagnosis, and receptor status was determined using immunohistochemistry with confirmatory fluorescence in situ hybridization for equivocal HER2 results when applicable. Recurrence patterns and survival outcomes were verified through patient charts, oncology follow-up databases, and national death registry records.

### 2.3. Treatment Modalities

Treatment decisions were made according to contemporaneous institutional practices and multidisciplinary tumor board recommendations, reflecting evolving standards over the 33-year study period. Surgical management primarily consisted of modified radical mastectomy, while breast-conserving surgery was performed in selected patients with early-stage disease or significant comorbidities. Adjuvant chemotherapy regimens included CMF in earlier decades and anthracycline or anthracycline/taxane based combinations in later years, whereas neoadjuvant chemotherapy was administered to patients with locally advanced tumors when deemed appropriate. Radiotherapy was delivered according to standard indications, typically following mastectomy in node-positive or locally advanced disease or after breast-conserving surgery. Endocrine therapy predominantly tamoxifen was recommended for all patients with hormone receptor-positive tumors unless contraindicated. HER2-positive patients were evaluated for trastuzumab eligibility in the later years of the cohort, and trastuzumab-based treatment was administered when available and clinically appropriate.

### 2.4. Outcome Definitions

The primary outcomes of the study were disease-free survival (DFS) and overall survival (OS). DFS was defined as the time from the date of pathological diagnosis to the first documented event of locoregional recurrence, distant metastasis, contralateral breast cancer, or death from any cause, whichever occurred first. Patients who remained alive and free of recurrence were censored at the date of last clinical follow-up. OS was defined as the interval from diagnosis to death from any cause, with survivors censored at last known contact. Recurrence patterns (locoregional, distant, or multiple-site involvement) were determined using clinical evaluations, radiologic imaging, and pathology reports. For patients with incomplete documentation of cause of death, national death registry records were reviewed to ensure accuracy and completeness. All dates were verified through institutional oncology information systems to minimize classification errors.

### 2.5. Statistical Analysis

All statistical analyses were conducted using SPSS software (version 23.0; IBM Corp., Armonk, NY, USA). Categorical variables were summarized as frequencies and percentages, while continuous variables were presented as medians with ranges. Age was incorporated as an a priori confounder and included in all multivariable Cox regression models irrespective of univariable significance. Comparisons between groups were performed using the chi-square or Fisher’s exact test for categorical variables and the Mann–Whitney U test for continuous variables, where appropriate. The proportional hazards assumption was assessed using Schoenfeld residuals and log–log survival plots. Survival outcomes, including disease-free survival (DFS) and overall survival (OS), were estimated using the Kaplan–Meier method, and differences between subgroups were evaluated with the log-rank test. Cox proportional hazards regression models were applied to identify predictors of DFS and OS, first using univariable analysis and subsequently incorporating significant variables (*p* < 0.05) into multivariable models to determine independent prognostic factors. Hazard ratios (HRs) with corresponding 95% confidence intervals (CIs) were reported for all regression analyses. All statistical tests were two-sided, and a *p*-value < 0.05 was considered statistically significant.

## 3. Results

### 3.1. Patient Characteristics

A total of 222 men with non-metastatic breast cancer were included in this study. The median age at diagnosis was 61 years (IQR: 53–71), and 27.9% of patients were aged ≥70 while 14.4% were younger than 50 years, indicating a predominantly older population. The median follow-up was 59.5 months (IQR: 32–104; range, 1–275). The majority of patients had invasive ductal carcinoma (91.9%), whereas papillary (3.1%), mucinous (1.4%), and mixed histologies were less common. High-grade tumors (grade 3) comprised 25.7% of cases. Tumor size distribution showed that 28.8% had T1, 40.5% T2, and 30.6% had T3–T4 disease. Axillary lymph node involvement was present in 57.7% of patients, and 34.2% of these had ≥4 positive nodes, indicating a substantial nodal disease burden. ER positivity was observed in 88.0% of the cohort, PR positivity in 82.3%, and HER2 overexpression/amplification in 22.8% among the 180 patients with available data. Triple-negative tumors accounted for 4.5% of cases (8 patients). Laterality was nearly balanced, with 51.8% left-sided and 48.2% right-sided tumors. Comorbidities were present in 31% of evaluable patients, most commonly hypertension, diabetes, and ischemic heart disease (Table 1).

All multivariable models were re-run with age forced into the model as an adjusted covariate, and assessment of the proportional hazards assumption demonstrated no significant violations.

### 3.2. Treatment Modalities

Among the 222 patients in the study cohort, modified radical mastectomy (MRM) constituted the major surgical approach and was performed in 84.2% of cases, whereas breast-conserving surgery was used in a small minority of patients with early-stage tumors or significant comorbidities. Axillary staging primarily consisted of axillary lymph node dissection (ALND), performed in 71.2% of patients, while sentinel lymph node biopsy (SLNB) alone was applied in 11.3%. A total of 158 patients (75.2%) received neo- and/or adjuvant chemotherapy, and chemotherapy use was significantly more common in younger patients (≤60 years: 88.8% vs. >60 years: 62.6%; *p* < 0.0001) and in those with lymph node–positive disease (87.2% vs. 68.1% in node-negative; *p* = 0.001) (Table 2).

Chemotherapy regimens evolved over time: earlier in the cohort, CMF represented the predominant regimen (10.2%), whereas anthracycline-based regimens (48.8%) and sequential anthracycline–taxane combinations (33.1%) became standard in later decades. Neoadjuvant chemotherapy was administered to 7.2% of patients (*n* = 16), mostly those with bulky T3–T4 tumors or clinically positive nodes. Radiotherapy was administered to 43.7% of all patients, primarily for high-risk pathological features such as ≥4 positive nodes, T3–T4 tumors, or close/positive surgical margins. Endocrine therapy was given to 79.5% of the overall cohort and to 86% of hormone receptor–positive patients, with tamoxifen being the predominant agent; aromatase inhibitors were reserved for patients with contraindications to tamoxifen or advanced age. HER2 testing was available in 180 patients, among whom 22.8% were HER2-positive; however, only 12 of these patients received trastuzumab-based therapy due to limited drug availability during earlier periods of the study.

Treatment intensification patterns reflected stage-dependent practice: patients with stage III disease were more likely to receive chemotherapy (86.5% vs. 68.6% in stage I–II; *p* = 0.003) and radiotherapy, consistent with contemporary guidelines and multidisciplinary recommendations.

### 3.3. Recurrence Patterns and Survival Outcomes

**Figure 1:**
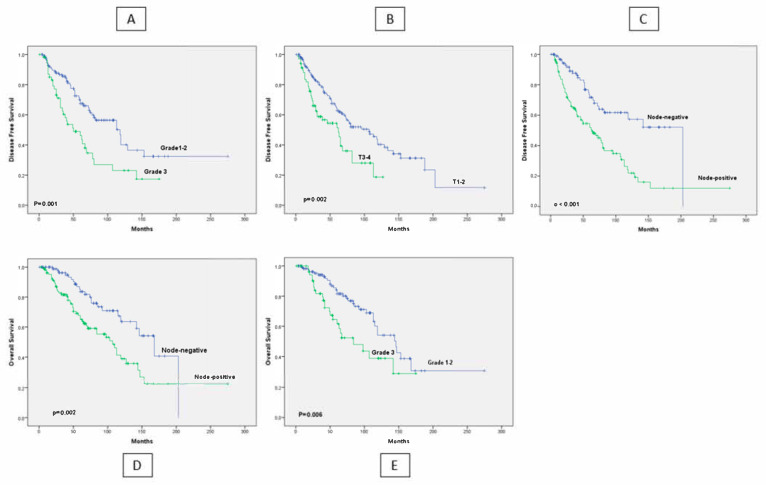
Disease-Free and Overall Survival Curves. Kaplan–Meier curves showing (**A**) DFS by tumor grade, (**B**) DFS by T-stage, (**C**) DFS by lymph node status, (**D**) OS by lymph node status, and (**E**) OS by tumor grade.

The median follow-up duration was 59.5 months (IQR: 32–104). A total of 82 patients (36.9%) experienced disease recurrence, and recurrence was significantly associated with higher T stage, lymph node positivity, high tumor grade, and the absence of adjuvant chemotherapy according to univariate analyses (*p* < 0.001 for all) (Table 3, Figure 1). Among the 79 patients with a documented cause of death, 52 (65.8%) died due to breast cancer.

Higher T-stage, lymph node positivity, and grade 3 tumors were associated with significantly worse survival outcomes (Figure 1).

In the univariate Cox regression analyses, advanced T stage, lymph node positivity, and high tumor grade were all significantly associated with inferior DFS and OS. Specifically, T3–T4 tumors showed a higher risk of recurrence (HR range: 1.6–2.1) and mortality (HR range: 1.9–2.1) compared with T1–T2 disease. Lymph node-positive patients demonstrated a 2.0–2.5-fold increased risk of recurrence and approximately a 1.8–1.9-fold increased risk of death. High-grade tumors were also associated with substantially worse outcomes, with HRs of 2.0–2.3 for DFS and 1.9–2.0 for OS. Other variables—including hormone receptor status, HER2 expression, surgery type, and adjuvant endocrine therapy—did not demonstrate statistically significant associations with survival in the univariate analyses.

The most common first sites of recurrence were bone (27.8%), lung (24.1%), and locoregional regions (16.5%), while 22.8% of relapsing patients presented with multisite progression. A total of 79 patients (35.6%) died during follow-up, of whom 52 (65.8%) were attributed to breast cancer–related causes. Median disease-free survival (DFS) for the entire cohort was 77 months (95% CI: 55.2–98.7), and median overall survival (OS) was 119 months (95% CI: 93.1–144.6). In subgroup analyses, DFS and OS were significantly shorter in patients with T3–T4 tumors, ≥4 positive lymph nodes, high-grade disease, and in those not receiving adjuvant chemotherapy or radiotherapy. For example, patients receiving adjuvant chemotherapy had a significantly lower risk of recurrence in node-positive disease (DFS HR: 0.58; 95% CI: 0.37–0.90) and also demonstrated an OS benefit (HR: 0.53; 95% CI: 0.32–0.88) compared with those who did not receive chemotherapy (Table 2).

Similarly, among patients with high-grade tumors, adjuvant chemotherapy resulted in a 48% reduction in recurrence risk (DFS HR: 0.52; 95% CI: 0.30–0.90). Conversely, endocrine therapy showed a trend toward improved DFS but did not reach statistical significance. HER2-positive patients receiving trastuzumab (*n* = 12) had substantially lower recurrence rates (25.0% vs. 52.4%) and markedly improved OS compared with HER2-positive patients who did not receive trastuzumab. Collectively, these findings confirm that stage, nodal burden, histologic grade, and adjuvant systemic therapy remain the dominant determinants of survival in male breast cancer.

### 3.4. Prognostic Factors

In univariate analyses, advanced T stage, lymph node positivity, high tumor grade, and absence of adjuvant chemotherapy were all significantly associated with inferior DFS and OS (Table 4). Patients with T3–T4 tumors demonstrated markedly shorter survival, with a DFS hazard ratio (HR) of 2.11 (95% CI: 1.44–3.11) and an OS HR of 1.96 (95% CI: 1.29–2.98). Lymph node–positive disease increased the risk of recurrence nearly 2.5-fold (HR: 2.47; 95% CI: 1.66–3.69) and the risk of mortality almost 1.9-fold (HR: 1.89; 95% CI: 1.20–2.98). High-grade tumors were similarly associated with significantly worse outcomes, with HRs of 2.27 (95% CI: 1.44–3.58) for DFS and 1.96 (95% CI: 1.19–3.21) for OS.

In multivariable Cox regression analysis, lymph node positivity, high tumor grade, and absence of radiotherapy independently predicted reduced DFS, whereas age ≥60 years, high-grade disease, and T3–T4 stage were independent predictors of poorer OS (Table 4). Collectively, these findings indicate that tumor burden, nodal involvement, and histologic aggressiveness remain the dominant determinants of long-term prognosis in male breast cancer.

### 3.5. Impact of Adjuvant Therapies

Adjuvant chemotherapy provided a significant survival benefit across several clinically relevant subgroups, as demonstrated in Table 5. Among patients with lymph node–positive disease, chemotherapy reduced the risk of recurrence by 42% (DFS HR: 0.58; 95% CI: 0.37–0.90) and decreased mortality by 47% (OS HR: 0.53; 95% CI: 0.32–0.88), highlighting its effectiveness in patients with nodal involvement. In high-grade tumors, chemotherapy was similarly beneficial, resulting in a 48% reduction in recurrence risk (DFS HR: 0.52; 95% CI: 0.30–0.90).

Hormone receptor-positive patients also experienced improved DFS with chemotherapy (HR: 0.62; 95% CI: 0.40–0.95), although the OS benefit did not reach statistical significance. Patients with HER2-positive disease who received trastuzumab showed numerically lower recurrence rates and substantially improved OS compared with those who did not receive HER2-targeted therapy.

Collectively, these findings demonstrate that adjuvant systemic therapy particularly chemotherapy offers meaningful benefit in several high-risk groups and remains an essential component of treatment for non-metastatic male breast cancer.

### 3.6. Subtype-Specific Outcomes

Subtype analysis revealed that patients with ER-positive/HER2-negative tumors had the most favorable outcomes, representing the majority of the cohort and achieving the longest DFS and OS. Triple-negative cases, although comprising a small subset, demonstrated the highest recurrence rates and the poorest survival. Among HER2-positive patients, those who received trastuzumab experienced notably fewer recurrences and longer overall survival compared with untreated HER2-positive patients, confirming the benefit of HER2-directed therapy when available. Overall, biological subtype remained an important determinant of prognosis in male breast cancer.

## 4. Discussion

Male breast cancer remains a rare malignancy, accounting for approximately 1% of all breast cancers, and contemporary data continue to show that its clinical behavior is broadly similar to that of postmenopausal female breast cancer, although outcomes are often less favorable in men due to delayed diagnosis and higher stage at presentation [15]. In the present multicenter cohort, we observed survival patterns consistent with several large retrospective datasets, including the EORTC 10085/TBCRC/BIG/NABCG program and recent SEER-based analyses, both of which highlight T stage, nodal burden, and tumor grade as the principal prognostic determinants in MBC [4,5,6]. Similarly to previous Turkish and international series, more than 30% of our patients presented with T3–T4 tumors and nearly half with axillary metastases, reflecting the persistent challenge of late-stage diagnosis in men [7,8,16]. The strong adverse impact of nodal positivity and high-grade histology in our study aligns with earlier findings by Cutuli et al. and Yadav et al., confirming that tumor burden at diagnosis continues to outweigh treatment-related variables in predicting long-term outcomes [4,5]. Large-scale English registry data similarly show distinct long-term survival patterns in male breast cancer compared with females [17], supporting our finding that prognosis in men is primarily driven by tumor stage and biological features. Moreover, despite increasing use of taxane-based chemotherapy and trastuzumab in recent decades, our multivariate analysis demonstrated that conventional clinicopathologic features still dominate survival modeling, underscoring the limited availability of molecular prognostic tools and subtype-specific treatment data in MBC [6,9].

The patterns of systemic treatment observed in our study closely mirror the historical evolution of adjuvant therapy in male breast cancer. Earlier cohorts reported very low chemotherapy utilization, often ranging between 7% and 30%, largely limited to CMF-based regimens in node-positive disease [4,9]. Over the past two decades, the adoption of anthracycline- and taxane-containing regimens has steadily increased, with recent population-based analyses from the United States reporting adjuvant chemotherapy use in nearly half of stage I–III MBC patients [5,10]. Our cohort demonstrates an even more pronounced shift toward modern regimens, with more than 75% of patients receiving neoadjuvant or adjuvant chemotherapy likely reflecting improved access to medical oncology services, broader acceptance of guideline-concordant treatment for MBC, and extrapolation from female breast cancer data. Importantly, the survival benefit observed with chemotherapy in our high-risk subgroups (nodal positivity, high-grade tumors, and HR-positive disease) aligns with both SEER-based analyses and the limited prospective data available in male patients [10,11,12]. Our cohort demonstrated a HER2-positive rate of 22.8%, which is slightly higher than typically reported in Western series but consistent with data from several regional studies. Limited access to anti-HER2 therapy during the earlier years of our study likely contributed to suboptimal treatment delivery in eligible patients. Recent evidence further underscores the biological heterogeneity of HER2-negative tumors in men, with reports identifying both a distinct HER2-low subgroup [18] and considerable variation in HER2 pathway activation across large male cohorts [19], findings that are consistent with our observation that HER2 status represents an important prognostic and potentially therapeutic determinant in male breast cancer. These evolving data highlight the need for more refined molecular characterization in male breast cancer to guide optimal systemic therapy selection. While HER2-positive disease was relatively uncommon, trastuzumab administration was associated with markedly lower recurrence rates and longer survival in our dataset, paralleling findings from the EORTC 10085/TBCRC/BIG/NABCG initiative [6] and supporting the inclusion of anti-HER2 therapy when clinically feasible. However, historic limitations in drug availability and reimbursement policies, particularly in middle-income settings, restricted trastuzumab utilization in earlier decades of our cohort a pattern noted in other real-world MBC series from Europe and Asia [13,14]. Recent real-world data from the Turkish Oncology Group further support the effectiveness of CDK4/6 inhibitors in male patients with HR-positive/HER2-negative breast cancer [20], highlighting the importance of expanding access to modern targeted therapies in this population.

Our findings are consistent with the recent COBCG meta-analysis demonstrating that radiotherapy significantly improves locoregional control and survival in men—particularly in node-positive and other high-risk subgroups [21]. Our study also showed higher RT utilization among patients with adverse prognostic features, supporting its essential role within the multimodality management of male breast cancer.

Endocrine therapy remains the cornerstone of systemic treatment in male breast cancer due to the overwhelmingly high prevalence of hormone receptor positivity, a pattern consistently observed across multiple studies including our own cohort, in which nearly 90% of evaluable patients were ER-positive. Tamoxifen continues to be the most widely used endocrine agent, supported by retrospective evidence demonstrating reductions in recurrence and mortality; however, real-world adherence in men is often suboptimal. Several large datasets have reported discontinuation rates as high as 20–25%, largely due to treatment-related side effects such as decreased libido, hot flashes, mood alterations, and weight gain, which appear to be more burdensome for men than women [22,23,24]. This challenge is compounded by the limited evidence supporting aromatase inhibitors in men, where incomplete estrogen suppression and concomitant gonadal feedback mechanisms reduce efficacy unless combined with GnRH analogs. Our findings, similar to previous reports, indicate that endocrine therapy was widely administered but did not demonstrate a statistically significant impact on survival, likely reflecting issues of adherence, competing comorbidities in older patients, and the relatively small HER2-negative, endocrine only subgroup. Another persistent limitation in the management of male breast cancer is the absence of validated genomic assays such as Oncotype DX or MammaPrint, which are extensively used in female breast cancer to guide chemotherapy decisions. Although exploratory studies have suggested that genomic signatures may have prognostic value in men, current assays were developed and validated exclusively in women, and ASCO/NCCN guidelines do not recommend their routine use in male patients [25,26,27,28]. As a result, treatment decisions in our cohort consistent with clinical practice worldwide relied primarily on traditional clinicopathological factors such as tumor size, nodal involvement, and grade. The predominance of luminal tumors and the absence of a standardized genomic approach underscore the need for improved molecular characterization of male breast cancer to refine risk stratification and treatment personalization in the future.

Our findings contribute meaningful real-world evidence to a field where high-quality data remain limited due to the rarity of male breast cancer. With 222 patients treated over more than three decades, our study represents one of the largest multicenter datasets from a single country and provides a robust evaluation of how long-term changes in diagnostic and therapeutic practices have influenced survival. The strength of our work lies in its large sample size, detailed clinicopathological annotation, and comprehensive survival analysis, which allow for a nuanced assessment of the prognostic value of traditional factors such as tumor size, nodal status, and grade features repeatedly confirmed in major international datasets, including SEER and the EORTC 10085/TBCRC/BIG/NABCG program [4,5,6]. Moreover, by stratifying outcomes according to stage, biological subtype, and systemic therapy use, our study offers one of the most detailed examinations of adjuvant chemotherapy effectiveness in high-risk subgroups, complementing and extending prior reports by Cutuli, Yadav, and others [9,10,11,12]. Nonetheless, several limitations warrant consideration. As with most studies in MBC, the retrospective design introduces potential selection and information biases, particularly given the long study period during which diagnostic standards, pathology techniques, and treatment availability most notably trastuzumab and taxane-based regimens changed substantially. Earlier decades were characterized by limited HER2 testing and incomplete documentation of endocrine adherence, both of which may have influenced survival estimates. The lack of genomic assays, which are increasingly used to guide adjuvant therapy in women, also constrained treatment individualization in this cohort [29,30,31]. Despite these limitations, our study provides valuable insights into real-world treatment patterns and survival determinants and underscores the urgent need for prospective, male-specific clinical trials as well as molecular profiling initiatives that could refine risk stratification and optimize systemic therapy in future MBC management.

## 5. Conclusions

In this large multicenter cohort of men with non-metastatic breast cancer, traditional clinicopathological features—particularly tumor size, lymph node involvement, and histologic grade—remained the dominant determinants of long-term survival. Adjuvant chemotherapy provided meaningful benefit in high-risk subgroups, including node-positive, high-grade, and hormone receptor–positive disease, while trastuzumab markedly improved outcomes in HER2-positive patients when available. Despite substantial improvements in treatment over the past three decades, the persistently high proportion of advanced-stage diagnoses highlights ongoing challenges in early detection. Our findings underscore the need for increased clinical awareness, more consistent application of modern systemic therapies in men, and the development of male-specific prospective studies and molecular tools to better refine risk stratification and personalize treatment in this rare disease.

## Figures and Tables

**Table 1:** Patient’s and tumor characteristics and treatment modalities for entire group of patients.

	N (%)
Median Age (*n* = 222)	61 (29–91)
Histology (*n* = 222)	
Invasive ductal	204 (91.9)
Invasive lobular	4 (1.8)
Mucinous	3 (1.4)
Other	11 (5.0)
Tumor grade (*n* = 172)	
Grade I	11 (6.3)
Grade II	104 (60.4)
Grade III	57 (33.1)
ER status (*n* = 208)	
Positive	183 (88.0)
Negative	25 (12.0)
PR status (*n* = 197)	
Positive	163 (82.7)
Negative	34 (17.3)
HER2 status (*n* = 180)	
Positive	41 (22.8)
Negative	139 (77.2)
Tumor size (cm) *n* = 221	
T1–2	153 (69.2)
T3–4	68 (30.8)
Lymph node status (*n* = 203)	
None	93 (45.8)
1–3	53 (26.1)
4–9	37 (18.2)
>10	20 (9.9)
Stage (*n* = 214)	
I	35 (16.4)
II	83 (38.8)
III	96 (44.9)
Surgery (*n* = 222)	
Modified Radical Mastectomy	187 (84.2)
Other	35 (15.8)
Adjuvant Radiotherapy (*n* = 222)	
Yes	97 (43.7)
No	125 (56.3)
Neo/Adjuvant Chemotherapy (*n* = 166) *	
Anthracyclin based	81 (48.8)
Anthracyclin and a taxane	55 (33.1)
CMF	17 (10.2)
Trastuzumab plus chemotherapy	12 (7.2)
Docetaxel plus Cyclophosphamide	1 (0.6)
Adjuvant Hormonotherapy (*n* = 222)	
Yes	175 (78.8)
No	44 (19.8)
Type of hormonotherapy (*n* = 175)	
Tamoxifen	172 (98.3)
Aromatase inhibitors	3 (1.7)

* One patient received neoadjuvant CT and data of neoadjuvant regimen is missing.

**Table 2:** Patient’s and tumor characteristics of the study cohort according to adjuvant chemotherapy status.

	Chemotherapy *	No Chemotherapy	*p* Value
Age (*n* = 222)			<0.0001
≤60	95 (88.8)	12 (11.2)	
>60	72 (62.6)	43 (37.4)	
Histology (*n* = 222)			0.778
Invasive ductal	154 (75.5)	50 (24.5)	
Other	13 (72.2)	5 (27.8)	
Tumor grade (*n* = 172)			1
Grade I–II	89 (77.4)	26 (22.6)	
Grade III	44 (77.2)	13 (22.8)	
ER status (*n* = 208)			0.630
Positive	136 (74.3)	47 (25.7)	
Negative	20 (80.0)	5 (20.0)	
PR status (*n* = 197)			0.190
Positive	120 (73.6)	43 (26.4)	
Negative	29 (85.3)	5 (14.7)	
HER2 status (*n* = 180)			0.316
Positive	33 (80.5)	8 (19.5)	
Negative	99 (71.2)	40 (28.8)	
Tumor size (cm) *n* = 221			0.616
T1–2	114 (74.5)	39 (25.5)	
T3–4	53 (77.9)	15 (22.1)	
Lymph node involvement (*n* = 203)			0.001
Yes	95 (87.2)	14 (12.8)	
			
No	64 (68.1)	30 (31.9)	
Stage (*n* = 214)			0.003
I–II	81 (68.6)	37 (31.4)	
III	83 (86.5)	13 (13.5)	

* Adjuvant and/or neoadjuvant chemotherapy.

**Table 3:** Cox Regression Model for DFS and OS.

Cox Regression Model for DFS and OS				
	DFS, HR (95% CI)	*p*	OS, HR (95% CI)	*p*
Age				
≤60 >60	-	-	1.00	0.015
	-		2.000 (1.147–3.487)	
T stage				
T0–1–2 T3–4	1.00	0.072	1.00	0.020
	1.621 (0.957–2.745)		2.091 (1.126–3.883)	
Lymph node				
Negative Positive	1.00	0.009	1.00	0.056
	1.958 (1.183–3.241)		1.741 (0.985–3.077)	
TNM stage				
I–II III	1.00	0.931	1.00	0.158
	0.971 (0.502–1.878)		0.569 (0.261–1.244)	
Tumor grade				
1–2 3	1.00	0.002	1.00	0.029
	2.070 (1.295–3.310)		1.867 (1.067–3.265)	
Surgery type				
MRM Other	1.00	0.145	-	-
	1.768 (0.821–3.808)		-	
Adjuvant CT				
No Yes	-	-	1.00	0.914
	-		0.963 (0.483–1.920)	

**Table 4:** Prognostic factors that may affect the DFS and OS.

	DFS Median Months	*p*	OS Median Months	*p*
All patients	77 (55.3–98.7)		119 (93.3–144.7)	
Age (years, *n* = 222)				
≤60	80 (36.8–123.2)	0.185	168 (129.7–206.2)	0.002
>60	68 (50.6–85.4)		107 (65.7–148.2)	
Tumor grade (*n* = 172)				
Grade I–II	118 (87.3–148.7)	0.001	146 (117.1–174.9)	0.006
Grade III	50 (24.6–75.4)		84 (37.5–130.5)	
ER status (*n* = 208)				
Positive	74 (60.8–87.1)	0.107	115 (94.8–135.1)	0.026
Negative	134 (71.0–196.9)		168 (134.9–201.0)	
PR status (*n* = 197)				
Positive	72 (58.9–85.0)	0.335	115 (93.3–136.6)	0.444
Negative	108 (67.9–148.0)		127 (79.9–174.0)	
HER2 status (*n* = 180)				
Positive	74 (45.1–102.9)	0.180	98 (79.0–116.9)	0.304
Negative	77 (42.2–10.29)		127 (95.8–158.2)	
Tumor stage (*n* = 221)				
T1–2	107 (72.4–141.5)	0.002	144 (115.7–172.3)	0.006
T3–4	62 (41.3–82.7)		76 (54.7–97.3)	
Lymph node (*n* = 203)				
No	203 (NA)	<0.001	168 (130.8–205.2)	0.002
Yes	63 (39.7–86.3)		107 (81.7–132.3)	
Stage (*n* = 214)				
I–II	119 (61.4–176.6)	<0.001	146 (101.8–190.2)	0.093
III	63 (46.1–79.8)		107 (76.9–137.1)	
Surgery (*n* = 222)				
MRM	82 (57.3–106.7)	0.003	127 (101.7–152.3)	0.171
Other	40 (18.3–61.7)		65 (43.6–86.4)	
Adjuvant Radiotherapy				
Yes	72 (58.3–85.7)	0.245	119 (92.6–145.3)	0.429
No	108 (33.7–182.3)		115 (57.6–172.4)	
Neo-/Adjuvant Chemotherapy				
Yes	80 (48.9–111.1)	0.960	86 (20.9–151.0)	0.252
No	68 (55.2–80.8)		127 (94.0–159.9)	
Adjuvant hormonotherapy (*n* = 222)				
Yes	80 (49.6–110.3)	0.080	119 (35.5–184.4)	0.195
No	60 (19.1–100.8)		110 (31.2–188.7)	

**Table 5:** DFS and OS benefit of adjuvant chemotherapy according to tumor and treatment characteristics.

	DFS, Months (95% CI)			OS, Months (95% CI)		
	Non-Chemotherapy *n*:64	Chemotherapy *n*:158	*p*	Non-Chemotherapy *n*:64	Chemotherapy *n*:158	*p*
Age						
≤60	NR	80 (38.1–121.9)	0.831	NR	168 (94.4–241.5)	0.318
>60	63 (43.7–82.3)	74 (48.0–99.9)	0.575	67 (37.1–96.9)	113 (76.1–149.9)	0.250
T stage						
T0–1–2	80 (28.3–131.7)	107 (73.8–140.2)	0.674	85 (7.5–162.5)	146 (118.5–173.4)	0.040
T3–4	63 (4.9–121.1)	62 (38.1–85.9)	0.841	63 (24.1–101.9)	76 (55.1–96.8)	0.294
Lymph node						
Negative	NR	142 (50.2–233.7)	0.358	NR	168 (134.9–201.1)	0.822
Positive	22 (13.6–30.4)	73 (55.0–90.9)	0.006	29 (23.7–34.3)	110 (98.4–121.6)	0.002
TNM stage						
I–II	NR	119 (74.5–163.4)	0.865	85 (NA)	153 (134.4–171.6)	0.132
III	31 (0.0–75.6)	65 (42.1–87.9)	0.161	45 (0.0–93.8)	113 (94.8–131.2)	0.057
Tumor grade						
1–2	NR	113 (70.3–155.7)	0.129	144 (NA)	119 (86.6–151.3)	0.491
3	67 (11.4–122.6)	50 (24.9–75.1)	0.962	67 (24.7–109.2)	107 (44.4–169.6)	0.351
HR						
Negative	NR	134 (43.9–224.1)	0.703	NR	168 (145.2–190.8)	0.890
Positive	67 (42.8–91.2)	77 (63.3–90.7)	0.920	84 (55.2–112.8)	119 (101.5–136.5)	0.068
HER2						
Negative	69 (20.2–117.8)	77 (42.4–111.6)	0.707	144 (29.4–258.6)	127 (95.2–158.7)	0.275
Positive	27 (0.0–68.5)	74 (54.1–93.9)	0.229	47 (20.4–73.6)	103 (85.5–120.5)	0.023
Surgery type						
MRM	80 (28.3–131.7)	82 (52.8–111.2)	0.922	85 (16.9–153.1)	127 (96.4–157.6)	0.091
Other	35 (9.6–60.4)	40 (7.7–72.3)	0.674	40 (32.4–47.6)	65 (44.9–85.0)	0.272
Adjuvant RT						
No	80 (NA)	108 (26.5–189.4)	0.905	84 (NA)	115 (63.5–166.5)	0.329
Yes	63 (16.8–109.2)	77 (61.6–92.4)	0.194	85 (26.8–143.2)	127 (94.3–159.7)	0.067

## Data Availability

Data are available from the corresponding author upon reasonable request and with permission from the Institutional Review Board, due to ethical and institutional restrictions on patient-level clinical data.
